# Defining Information Quality Into Health Websites: A Conceptual Framework of Health Website Information Quality for Educated Young Adults

**DOI:** 10.2196/humanfactors.6455

**Published:** 2017-10-06

**Authors:** Donghua Tao, Cynthia LeRouge, K Jody Smith, Gianluca De Leo

**Affiliations:** ^1^ Medical Center Library Saint Louis Univesity St. Louis, MO United States; ^2^ Department of Information Systems and Business Analytics College of Business Florida International University Miami, FL United States; ^3^ Department of Health Sciences and Informatics Doisy College of Health Sciences Saint Louis University St. Louis, MO United States; ^4^ Department of Clinical and Digital Health Sciences College of Allied Health Sciences Augusta University Augusta, GA United States

**Keywords:** consumer health information, World Wide Web, Internet, information services, quality control, young adults, evaluation studies as topic, medical informatics

## Abstract

**Background:**

Today’s health care environment encourages health care consumers to take an active role in managing their health. As digital natives, young educated adults do much of their health information management through the Internet and consider it a valid source of health advice. However, the quality of information on health websites is highly variable and dynamic. Little is known about the understandings and perceptions that young educated adults have garnered on the quality of information on health websites used for health care–related purposes.

**Objective:**

To fill this gap, the aim of this study was to develop a conceptual framework of health website information quality with quality dimensions (ie, criteria) and associated quality drivers (ie, attributes) specified in the context of young educated adults’ use of health websites for health care–related purposes. This aim was achieved by (1) identifying information quality dimensions of health websites from the perspective of young educated adults; (2) identifying the importance ratings of these quality dimensions; and (3) constructing a framework of health website information quality with quality dimensions and associated drivers specified in the context of young educated adults’ use of health websites for health care–related purposes.

**Methods:**

The study employed both qualitative and quantitative methods. Methods included semistructured group interviews and an individual quality assessment exercise grounded in visiting various websites and responding to Likert scale questions regarding the importance ratings of information quality dimensions and open-ended questions with specifying website quality drivers. Study participants included junior and senior undergraduate and graduate students in business, allied health, and public health majors. Qualitative, open-coding procedures were used to develop the conceptual framework reflecting the participants’ means of assessing information quality on health websites.

**Results:**

Five dimensions of information quality for health websites were identified: Completeness of information, Understandability of information, Relevance of information, Depth of information, and Accuracy of information. Completeness of information and Understandability of information were rated as the two most important quality dimensions by the study participants. Results indicated that these five information quality dimensions for health websites were supported by the following main driver themes: Content, Design, Links, Consumer resources, Search functionality, Supporting references, User focus, Content FAQ, Open access, Policy statements, and Site performance.

**Conclusions:**

This study contributes to the literature by developing a health website information quality conceptual framework with quality dimensions and associated drivers specified for a young educated adult population. The detailed quality drivers supporting the corresponding quality dimensions provide a rich picture of young educated adults’ perceptions on health website information quality. This framework can be used to guide the development of health websites, as well as the foundation for a means to evaluate health information from existing health websites with young educated adults as the target audience.

## Introduction

### Background

Today’s health care environment encourages health care consumers (patients and caregivers) to take an active role in participating in their health care–related decision making and managing their own health [1]. Literature indicates that most consumers prefer to receive information about their illnesses and treatment options from multiple sources, including health care providers, other patients, and the Internet [2,3]. Health information on the Web increasingly plays an important role for consumers making a health care decision [4-6]. A 2013 Pew Project revealed [2] that 59% of the adults in the United States have looked on the Web for health information, with 6.75 million health care–related searches being performed per day [7] and 35% of people use the Web-based health information to make diagnoses but only half of them check with medical professionals [2]. Erroneous and misleading health information on the Web increases the risks of wrong self-diagnosis, damaging treatment attempts, and delaying or canceling doctor visits [2]. Given the magnitude of the amount and use of health information on the Web and its significant impact on consumers’ health care decisions, as well as their overall approach to maintaining health, it is imperative that health websites provide consumer-perceived quality health information used for health care consumers making informed health care decisions and other health care–related purposes.

The study of health information quality is somewhat complicated because of various perspectives of defining and measuring information quality [8-14]. Past systematic reviews on the quality of health information for consumers on the Web [10,11,13] acknowledged the complexity of this concept because of the existence of the large number of criteria and different ways to categorize them. Reviews also recognized the lack of conceptual clarity regarding the consensus on what constitutes information quality and what the major dimensions and attributes are. Among the studies that have explored information quality from a health care consumer’s perspective [7,9,15-17], few have made efforts to extensively define and specify the quality dimensions and the underlying attributes, which results in a lack of clarity regarding consumers’ perceptions on information quality. In this regard, construct development is needed to decompose and better understand the construct of information quality from the perspective of those likely to use technology for health care–related purposes.

The concept of information quality is also complex in the eyes of health care consumers [10,12] and goes well beyond an assessment of information accuracy [8-10]. Although the involvement of health experts will enhance the accuracy of health information, reliance on the perspective of health experts can be problematic. Health care consumers seek and appraise information differently from experts [15] in specifying the different quality dimensions and associated attributes that define information quality. Moreover, health care consumers’ perceptions of information quality impact the perceived usefulness and ease of use of a health information system, which further impacts their use and continued use of the system [18-23]. To design and develop a health website that better meets the expectations of health care consumers, further research is needed to conceptualize information quality from health care consumers’ perspectives. A more complete understanding of this perspective may provide guidance for user-centered websites that can help consumers seek and evaluate health information, and thus, assist with their self-care and other health care–related purposes.

We focus this study of health information quality on college-educated young adults to reflect the demographics of health website users as among the most likely to seek and depend on health information on the Web [2,24,25]. As digital natives, young adults can exploit high levels of interactivity and personalization features available in the health websites that allow them to take advantage of using health information on the Web for health care–related purposes [26]. It is conceivable that this target group would be Internet savvy for discerning health information quality on a website. However, studies to date have not specified the quality of health information for websites for this consumer group.

The goal of this study was to develop a health website information quality conceptual framework with quality dimensions (ie, criteria) and associated quality drivers (ie, attributes) specified in the context of young educated adults’ use of health websites for health care–related purposes. We use general model structures of system and service quality found in the information system and marketing literature as a starting point to explore the dimensions of information quality, as well as the attributes that drive each of the information quality dimensions. The process we take to attend to this goal involves (1) identifying information quality dimensions of health websites from the perspective of young educated adults, portrayed to be among the most active technology health care consumers; (2) establishing the importance ratings of the identified health website information quality dimensions; and (3) constructing a health website information quality framework with quality dimensions and associated drivers deemed relevant by young educated adults.

### Young Educated Adults as Health Care Consumers

Young adults (in the age range of 18-26 years) are seen as generally healthy. Yet, they face challenges to keep healthy while reducing the risk of developing chronic conditions. Mental health, substance abuse, homicides, suicides, and motor vehicle accidents are all areas of concerns that impact the overall health and life of a young adult. These issues and challenges make young adults search for health-related information via the Internet to cope with health-related concerns and stresses [27-29]. Young adults search health information for various purposes, such as learning about health conditions, seeking online support, looking for treatment options, and prevention and screening information [2,30,31], or see the Internet as an acceptable resource that offers *anonymized* information or support for sensitive conditions or symptoms [32].

As reported by the 2015 Pew Research Center Report, “for some groups, especially young adults, those with high levels of education, and those in more affluent households, internet penetration is at full saturation levels” [24]. The study found that 93% of young adults (in the age range of 18-29 years) have remained the most likely to go on the Web, even as the Internet population has grown and even with documented larger increases in certain age cohorts (eg, adults aged 65 years and older) [33]. Research indicates that young adults trust the information on the Web and consider the Internet as a valid source of health advice [30,34], which calls for the necessity of not only ensuring the accuracy of health information on the Web but also providing content and design that allows users to cognitively and perceptually discern information quality.

Studies found that younger adults do much of their health information management through the Internet and that those groups most likely to have done so are between the ages of 18 to 29 years, women, and college graduates [2,24,25]. Previous studies examining the use of the Internet for health information have focused on populations of interests, including healthy volunteers [7,9], clinicians [8,35], caregivers [16], and adult patients [17], with age ranging from 19 to over 65 years. Yet, few studies have focused on the young adult population [36,37]. How young adults group perceives the quality of health information from health websites remains unclear.

### Information Quality

Information quality has been defined as *fitness to use* [38]. The DeLone and McLean Information System Success Model [39] demonstrated that information quality is an antecedent to system use and user satisfaction that lead to system benefits. As the most frequently tested model in the information system literature, the Technology Acceptance Model (TAM) indicates that perceived usefulness is important regarding the attitude toward technology and the ultimate behavioral intention [19], and perceived information quality is partial perceived usefulness. Empirical studies have examined the relationship between information quality dimensions and higher level evaluations. For example, several studies applied the System Success Model, TAM, and Web service quality models to successfully demonstrate the connections between perceived information quality, perceived usefulness, and intention to use or actual use [16,18-23,39-43]. A study investigating the trust factor in consumers’ decisions regarding whether to use Web-based health advice indicated that credibility of information and personalization of content predicted *selection* (trust) of advice sites [24]. Fewer studies have focused on the linkage of quality drivers to dimensions.

**Figure 1:**
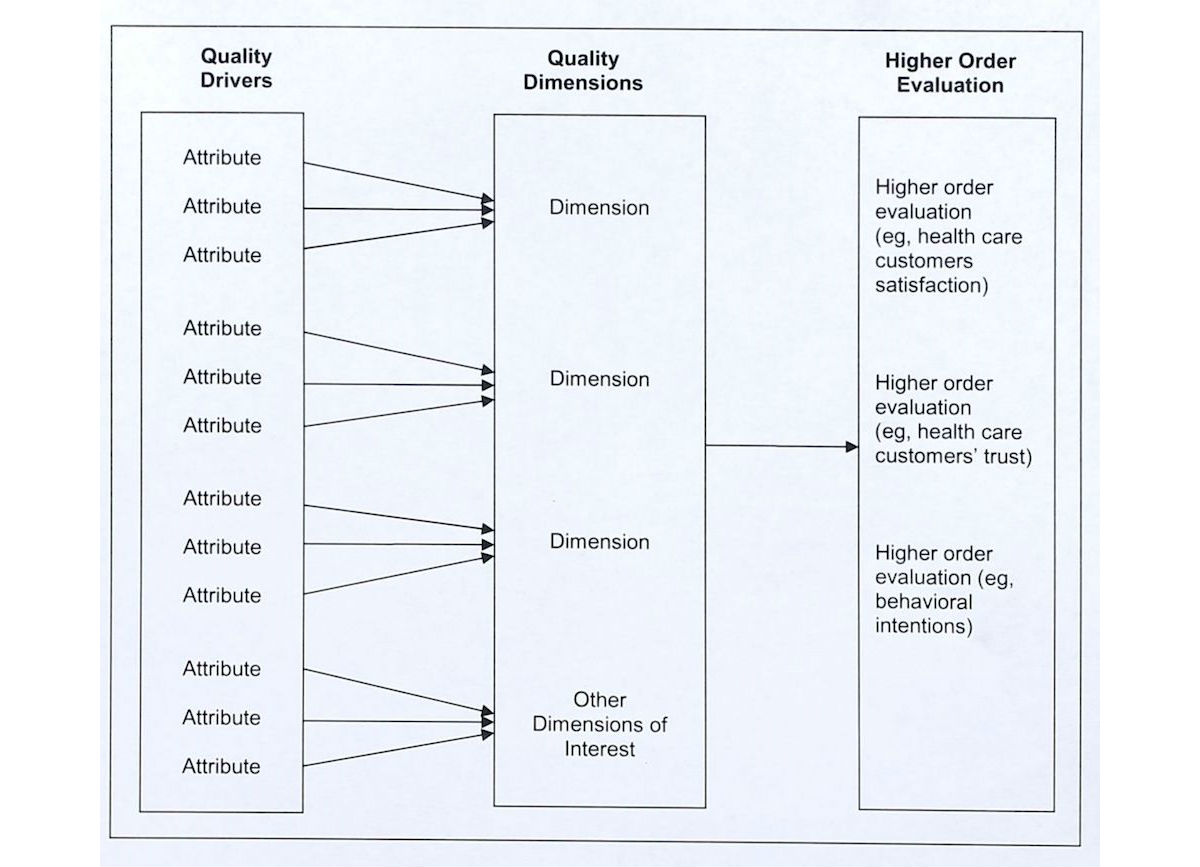
General model structure on three levels of website evaluation and their relationships.

Indeed, both information system and marketing literature provide conceptual models that exhibit the general structure of linking objective or perceived quality attributes (ie, drivers) to perceived quality dimensions and subsequently to other higher level evaluations of technology success [19,39,40]. Figure 1 summarizes this general structure and draws attention graphically to the distinctions and relationships between the concept of drivers and dimensions [19,39,40]. This distinction helps to clarify the health information quality construct and proposes potential causal relationships. As indicated, the leftmost box of Figure 1 contains quality attributes (ie, drivers), which may be objective or perceptual. The middle box represents the model of system quality dimensions (ie, criteria). Finally, the rightmost box contains elements such as overall customer satisfaction, customer trust, and behavioral intentions (eg, intent to use the system). We use this general model structure as a starting place and adapt it to the context of health care where young educated adults search for health information from health websites for health care–related purposes.

#### Health Information Quality Dimensions Recognized in the Literature

Information quality is recognized as a multidimensional concept [44]. Terms such as *quality dimensions* [9,45] and *criteria* [10,11] have been used to reference the multidimensional nature of website quality. We define quality dimensions for purposes of this study as “abstract rules by which the quality of information is judged,” which aligns with the definition used in a recent systematic review on health information quality criteria [13,46]. For this study, information quality dimensions are quality characteristics manifested in health information.

Health information quality evaluation by consumers, as part of health website quality evaluation, has received considerable research attention and resulted in notable literature reviews. Kim et al [11] identified content, design and aesthetics, disclosure, and currency as information quality dimensions. Eysenbach [10] and Seidman [12] found that the most frequently used dimensions to evaluate health information quality included accuracy, completeness, readability, design, and a series of technical criteria (eg, disclosure, reference provided, and internal search engine present). Neither of these reviews identified dimensions specific to particular consumer groups. Zhang et al [20] presented 11 dimensions grouped in three categories: (1) Substance criteria included accuracy and completeness; (2) formality criteria included currency, credibility, and readability; and (3) design criteria included accessibility, aesthetics, navigability, interactivity, privacy and data protection, and cultural sensitivity. In addition to the dimensions summarized in the above three reviews, quality criteria, such as accuracy [8-10,13], comprehensiveness [8-10,13,41], credibility [10,13,41,47], authority [13,48-50], understandability [10,15,51], relevance [15,51], and currency [11,13,15] have been used to evaluate health website information quality in many empirical studies. Summatively, research has produced differences as well as commonalities in quality dimensions and various ways to categorize these dimensions; this reflects the complexity of the concept and the lack of consensus on defining information quality [13]. There are a number of potential reasons for these variances, including the method of constructing the list, prospectively identifying a limited number of potentially relevant dimensions to study, and efforts to overgeneralize and aggregate studies targeting different user groups. Furthermore, none of these quality dimensions were designated to the young educated adult population.

The ability to achieve ideal levels of information quality may be limited by resources (time and money), which makes understanding the rating of information quality dimensions to be useful in feature and content trade-off situations. Furthermore, rating relevant dimensions provides research insight into the evaluation process that consumers exercise in assessing information quality. Only a few studies investigated the priority of quality dimensions, and these studies indicate that not all quality dimensions are equally weighted in the health care consumers’ evaluation process. Stvilia et al found that health care consumers rated information quality dimensions in the following order based on a 5-point Likert scale: (1) accuracy, (2) completeness, (3) authority (reputation), (4) usefulness, and (5) accessibility [9]. In contrast, Stanford and colleagues found currency of the information is valued most by general health care consumers [52]. The conflict in findings of the health care consumer’s rating among studies may, in part, be attributed to the limitations in the scope of information quality dimensions used in individual studies, which supports the importance of developing a broad list of relevant information quality dimensions to obtain a more complete picture.

#### Health Information Quality Drivers Recognized in the Literature

Information quality dimensions offer some insight but not sufficient guidance to the content and design features that trigger user assessments of information quality. We define information quality drivers as the observable attributes that consumers expect or look for when they evaluate health website information quality [13]. Association of these drivers with the information quality dimensions they support helps to relate concrete features of the abstract quality dimensions. Studies that aligned with the definition of drivers used in this study indicate that consumers determine health information quality by looking for quality drivers such as owners of the website, source of the content, author’s credentials, additional source of support (eg, links), disclosure information, quality seal and third-party endorsement, including government agencies or professional associations, and so on [6,9,10,13,16,35]. These quality drivers serve as clues to whether heath information contained on a health website meets a given quality dimension criterion. From this perspective, quality drivers (we use the term quality drivers henceforth) are akin to *quality indicators* [10,15,46], *quality markers* [9], and *surrogates* [53] identified in past studies, which have used both quantitative and qualitative methods to dig more deeply into the underlying meaning of quality dimensions. These quality drivers serve as signals to the visitor of the quality of information contained on health websites. Consumers evaluate information quality by looking for these signals [9].

Identifying and distinguishing information quality drivers for health websites from existing literature is somewhat challenging. The confusion of classifications of quality dimensions and drivers presented in the literature creates some difficulties in discerning the three levels of the evaluation (see Figure 1) for health information quality. For example, *disclosure* was classified as one quality criterion at the dimension level in some studies [11], whereas it was recognized as a quality driver when consumers judge the credibility of information in other studies [9,13,35]. Eysenbach et al summarized a list of drivers, indicating how information is presented on the website, but those drivers were classified as criteria at the dimension level in the study [10]. Similarly, Bernstam et al used 15 quality drivers to evaluate information quality from breast cancer websites but labeled them as technical quality criteria [35]. Moreover, we found that individual quality attributes were grouped together representing the same aspects of website design (eg, identity, purpose, content, design, user-feedback, and privacy), and these quality groups were named as criteria [14] or constructs [35], which mixed up the levels of quality dimensions and drivers.

These variations of the classifications and naming across previous studies challenge defining the information quality construct and identifying the associations of quality dimensions with concrete quality drivers perceived by health care consumers in the health website environment. It is difficult to discern whether individual drivers or classes of similar drivers (referred to as themes) contribute to multiple information quality dimensions. Some studies proposed [6] or tested the association between quality drivers and the corresponding quality dimensions but failed [35]. Similar to the value of rating dimensions, identifying drivers that contribute to multiple quality dimensions facilitates prioritization and can highlight key tangible factors in the user evaluation process.

### Research Questions

It is not surprising that Zeithami et al [40] suggested that future research focus on investigating the importance of different dimensions and perceptual attributes or drivers essential to electronic service quality and that Bliemel and Hassanein called for more research on consumer perspectives regarding health information quality evaluation [54]. Research is needed to discern how health care consumers understand and perceive health website information quality dimensions and the underlying attributes of each relevant dimension [55].

In response to the aforementioned issues and research gaps, the overall goal of this mixed-method study was to conceptually develop dimensions of the information quality concept and the associated quality drivers of each dimension in the context of young educated adults searching for health information from health websites for health care–related purposes.

To attend to these study purposes, we propose to answer the following research questions:

1. What dimensions (from a health care consumer perspective) comprise appropriate criteria for the design and the evaluation of quality of information published on health websites?
2. How do health care consumers rate the importance of the quality dimensions identified for information quality of health websites?
3. What are information quality drivers for each individual dimensions of information quality from a health care consumer perspective?

We will address these questions using the general model structure of website evaluation and their relationships (see Figure 1) as a general guide to (1) identify dimensions of health website information quality from the perspective of health consumers, (2) assess the importance of each dimension, and (3) present a conceptual framework of health website information quality with quality dimensions and associated supporting drivers by grouping drivers with driver themes to facilitate a means to begin to discern commonalities across dimensions.

## Methods

### Data Collection

This study employs a mixed-method design that includes quantitative (survey) and qualitative methods (group interview and open-ended website assessment exercise) to address the research questions. Mirroring past studies that explore the dimensions and factors of quality [45,56], we tapped into the knowledge of current and potential users of health websites using a user-centered approach that facilitated a ground-up conceptualization of information quality from the user perspective. To emulate current and potential users of health websites, our study participants are in the age range of 20 and 41 years and college educated (to stabilize education level among participants). Participants were recruited via class announcements and flyers. We obtained the approval from the institutional review boards at the institutes where the participants studied.

This approach was used to provide a comprehensive and relevant conceptual framework of information quality dimensions that tightly reflected the health care consumers’ perspective. The framework was constructed in two phases. Phase 1 involved developing a list of quality dimensions informed by a consumer perspective, and phase 2 involved an exercise to (1) validate and prioritize the quality dimensions identified in phase 1 and (2) discern quality drivers for each of these dimensions to specify the health website information quality framework.

#### Determining Dimensions

In phase 1, we determined health website information quality dimensions of interest (level 2 of our framework) through four semistructured group interviews with junior and senior undergraduates and graduate students. Group interview was chosen to allow building and inspiration from the comments of others in efforts to develop a comprehensive list of dimensions [57]. Most student participants were within health care domains (but not engaged in direct patient care), as well as business domains. Approximately 10 students participated in each interview. Participants confirmed that they had visited health websites before phase 1 participation. In addition, the interview protocol included the question “What health websites are you most familiar with?” to further ensure all participants had direct experience with health websites being explored, could ground their responses, and to inspire candidate websites to be seeded in phase 2 of data collection.

The remaining interview questions were inspired by the higher order constructs presented in Figure 1. The protocol included questions and probes that attempted to cover all relevant dimensions of the concept of health information quality. The participants responded to the primary questions, “What quality dimensions of a health website would lead to... (1. visitor satisfaction, 2. promoting desired behaviors by the website sponsors, 3. visitor website loyalty, and 4. visitor trust)?” Participants in the group interviews were asked to address these questions from their general knowledge based on personal experience, the experience of others, and other information sources. As the number of responses from the group diminished, a few probing questions asking the participants to consider various perspectives (well, sick, chronically ill, and had an injury) were introduced (eg, “if I were a...” patient and well-person looking for information) to ensure the group had exhausted their thoughts and to promote a comprehensive response. Interviewees were then asked to comment on the relevance of potential dimensions found in the literature that were not included in their responses, as a last measure to exhaust perspectives (note this literature included generalized reference to site visitors [10,47] as well as more specific patient populations [58,59]). We introduced data found from existing literature to ensure no key attributes were overlooked. Closing prompts directed participants to review the list of dimensions discussed by the group and inquired “anything else” and “is there anything missing” until it was clear that the group was saturated. There was increasing overlap and redundancy with prior groups in the dimensions identified for each subsequent group interview.

All dimensions suggested as relevant by any interview group were included in the cumulative list of dimensions for phase 2 of data collection. The research team reconciled conceptually redundant terms within and across interview groups. Furthermore, the team performed a literature review to determine whether the dimensions identified could be traced to prior literature (alignment of conceptual meaning). In such cases, where the dimension identified aligned with the conceptual meaning of terms found in past literature, further refinement of the term was done to facilitate connections between this study and prior research. The final, collective dimension list was a cross section of the dimensions identified by all four groups (see Table 1, which identifies the dimensions and provides connections to existing studies).

#### Determining Quality Drivers

Drawing on the dimensions provided by the foundational analysis, phase 2 consisted of a quality assessment exercise developed to determine underlying quality drivers. Junior and senior undergraduate and graduate students in colleges of business, allied health, and public health at two universities completed the exercise (198 students in total—92 students from the health-related domain and 106 from the business domain). No participants were health care practitioners. One university was in the Midwest of the United States and the other in the Eastern region of the United States.

As their first task, participants rated the general importance of each information quality dimension for health websites identified in phase 1 using a Likert scale on low importance to high importance scale of 1 to 5, with 1 anchored as: “I do not consider this characteristic at all in my assessment of this type of website”; and 5 anchored as: “This characteristic is very important to my assessment of this type of website.” The participants did not visit any websites as part of this assessment. We used basic statistics for importance ratings of information quality dimensions for health websites.

Task 2 required participants to identify quality drivers for each of the dimensions. To conceptually ground participants in the actual decision-making process of assessing information quality, participants visited two health websites (one seeded—Web MD and the second of their choice). Participant choices in their second site selection varied widely. By design, the order of visiting the two websites varied to reduce bias. Participants were asked to rate the websites with a focus on website context and the website quality decision-making process. Participants rated the two websites according to the identified importance dimensions. This rating was only used to stimulate thought and not as part of data analysis.

After the rating exercise, to gain insight into what drives the importance ratings of each quality dimension for health care websites, participants responded to a qualitative question of primary interest of this study, “What would cause you to rate a health website with a high score of 5 for name of quality dimension?” For example, “What would cause you to rate a health website with a high score of 5 for understandability?” Participants responded to the same questions for each dimension for two health websites. This was done to test the *within-subject* consistency in response to the assessment criteria.

### Data Analysis

We calculated descriptive statistics such as the mean and standard deviation of the responses to the general importance of information quality dimensions. To understand the total span of our data, we also calculated the minimum and maximum values.

Qualitative procedures were used to review the participants’ written commentaries to the open-ended questions asking them to explain what would cause them to rate a high score associated with five quality dimensions. Two researchers with expertise of different domains (information science and health information management) independently performed open coding [60] by identifying meaningful text from the responses that disclosed specific website drivers that would support each quality dimension. There was no predefined coding schema. As team members discovered new drivers associated with each quality dimension that did not map to the drivers they previously identified, they created a new code, a child code to the quality dimension, to explain a finding. Each coder independently reviewed and refined their code list containing detail drivers for each dimension.

A code reconciling process based on consensus was conducted to reach a stable list of drivers by integrating the perspectives of the 2 initial coders and a third member of the research team [61-68] for similar consensus-building approaches using investigator triangulation [69].

The third coder (representing the health information systems domain) who did not conduct individual coding work participated in the reconciliation process with the 2 open coders. The third coder reviewed the open coding performed by the previous 2 coders. With a third coder acting as referee, the coders reviewed and compared their resulting code lists to reconcile conceptually redundant code labels, to ensure adequate support existing for a code, to refine the labeling of resulting themes, and to harmonize the granularity of the codes. In cases where one coder identified a code not identified by the second coder, the team of three examined all the supporting text, working toward a reconciled agreement on whether the code was properly supported and should be represented in the health information quality framework as a detailed driver of identified quality dimensions. Such cases were a result of coder differences in granularity, which is when one coder created a broader code conceptualization than the other coder. When the expanded schemas involving each of the quality dimensions supported by detailed drivers appeared to become stable and three members reached consensus, the initial phase of data analysis was complete.

The 3 coders then performed axial coding, which is the process of relating codes to each other via a combination of inductive and deductive thinking [59] to group the resulting codes into quality driver themes that could be discussed across quality dimensions. The agreed upon themes among the 3 coders were defined as the quality driver categories (referred to as driver themes in the Results section). The final construction of the comprehensive framework of health website information quality consisted of the identified quality dimensions (presented with first letter capitalized), the quality driver themes for each dimension (presented with italicized and first letter capitalized), and supporting quality drivers (eg, codes)(presented with italicized only). We provide the differences in presentation to assist the reader in identifying the referenced level of the framework for each concept presented.

## Results

### Information Quality Dimensions and Importance Ratings for Health Websites

Five dimensions of information quality in the context of health websites emerged: (1) Accuracy of information; (2) Completeness of information; (3) Depth of information; (4) Understandability of information; and (5) Relevance of information. The definition of each dimension and the corresponding example studies are presented in Table 1.

Table 2 lists the importance of these quality dimensions across all participants. Completeness of information and Understandability of information were the two top dimensions perceived by study participants. These dimensions may serve as the foundation for health website sponsors and designers to consider in their website design and evaluation.

**Table 1:** Health websites information quality dimensions and their definitions.

Quality dimension	Definition
Accuracy of information	The degree of concordance of the information provided with the best evidence or with generally accepted medical practice [8-10,20]
Completeness of information	The proportion of priori-defined elements covered by the website; breath of information [8-10,20,24]
Depth of information	Level of information details [18,24,38]
Understandability of information	Readability with information in plain language containing statistics of text, explanations of medical language and acronyms, choice of display formats for numerical or graphical information, and clarity of images [10,11,20,37]
Relevance of information	Applicability of each item of content to potential users’ health situations, such as personalized health tools or age-specific information [11,37]

**Table 2:** Information quality dimension list and importance rating for health websites.

Dimension	N	Responses, n (%)						Median		Mean (SD^a^)
		1	2	3	4	5				
Completeness of information	196	0 (0.0)	0 (0.0)	1 (0.5)	21 (10.1)	174 (88.8)	5		4.883 (0.3382)	
Understandability of information	196	0 (0.0)	0 (0.0)	1 (0.5)	25 (12.8)	170 (86.7)	5		4.862 (0.3601)	
Relevance of information	195	0 (0.0)	1 (0.5)	9 (4.6)	49 (25.1)	136 (69.7)	5		4.641 (0.5958)	
Depth of information	196	0 (0.0)	1 (0.5)	12 (6.1)	57 (29.1)	126 (64.3)	5		4.571 (0.6325)	
Accuracy of information	159	0 (0.0)	8 (4.0)	18 (11.3)	21 (13.2)	112 (70.4)	5		4.491 (0.8850)	

^a^SD: standard deviation.

### Drivers of Information Quality Dimensions for Health Websites

**Figure 2:**
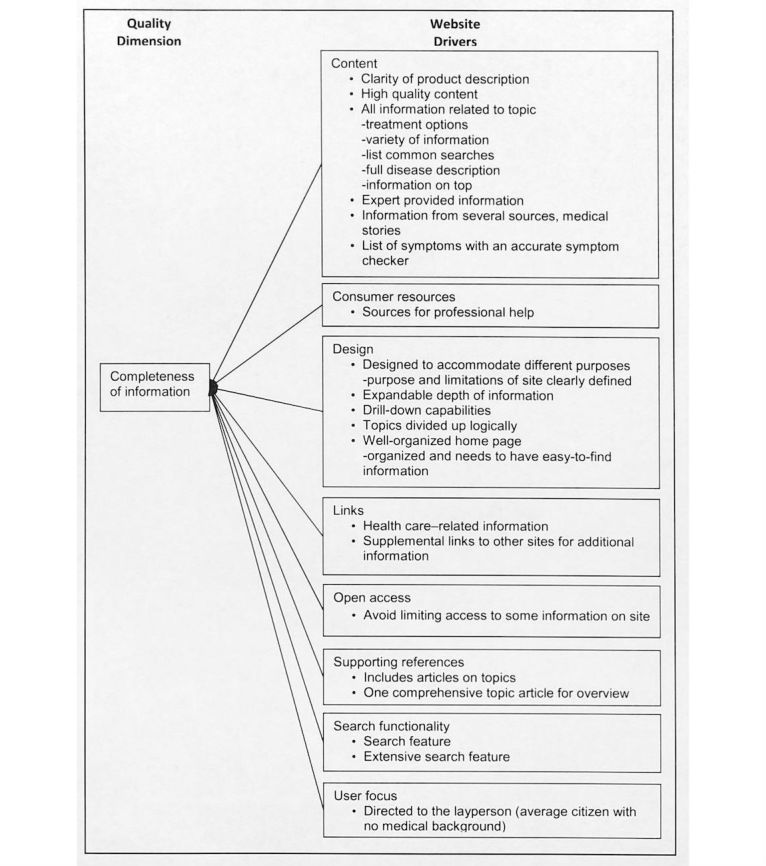
Quality driver themes and detailed drivers for completeness of information on health websites.

**Figure 3:**
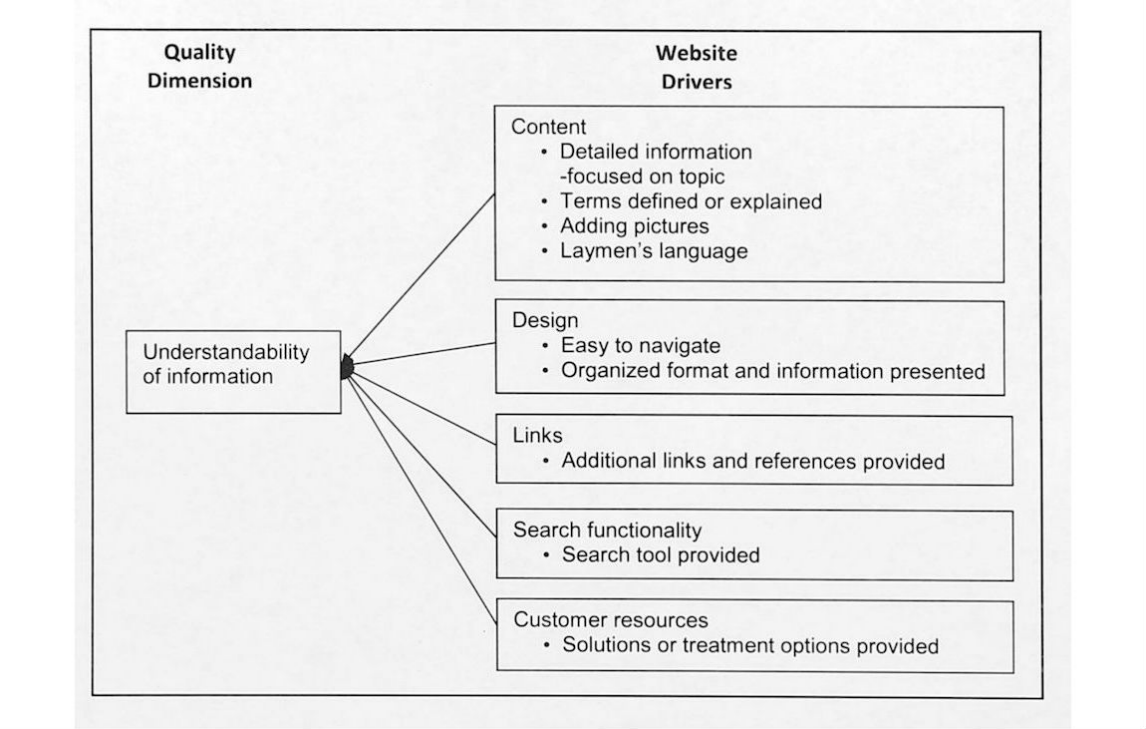
Quality driver themes and detailed drivers for understandability of information on health websites.

**Figure 4:**
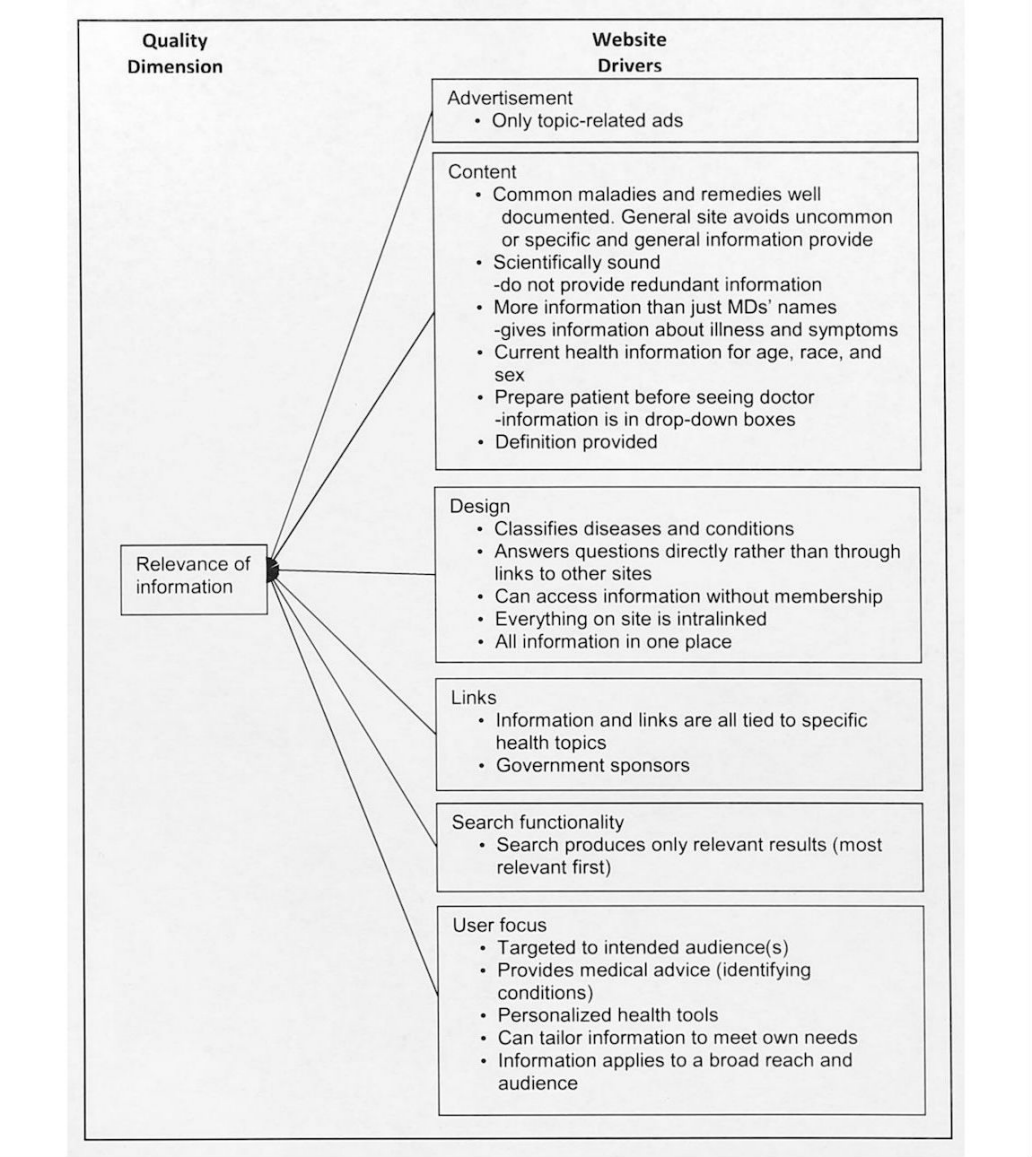
Quality driver themes and detailed drivers for relevance of information on health websites.

**Figure 5:**
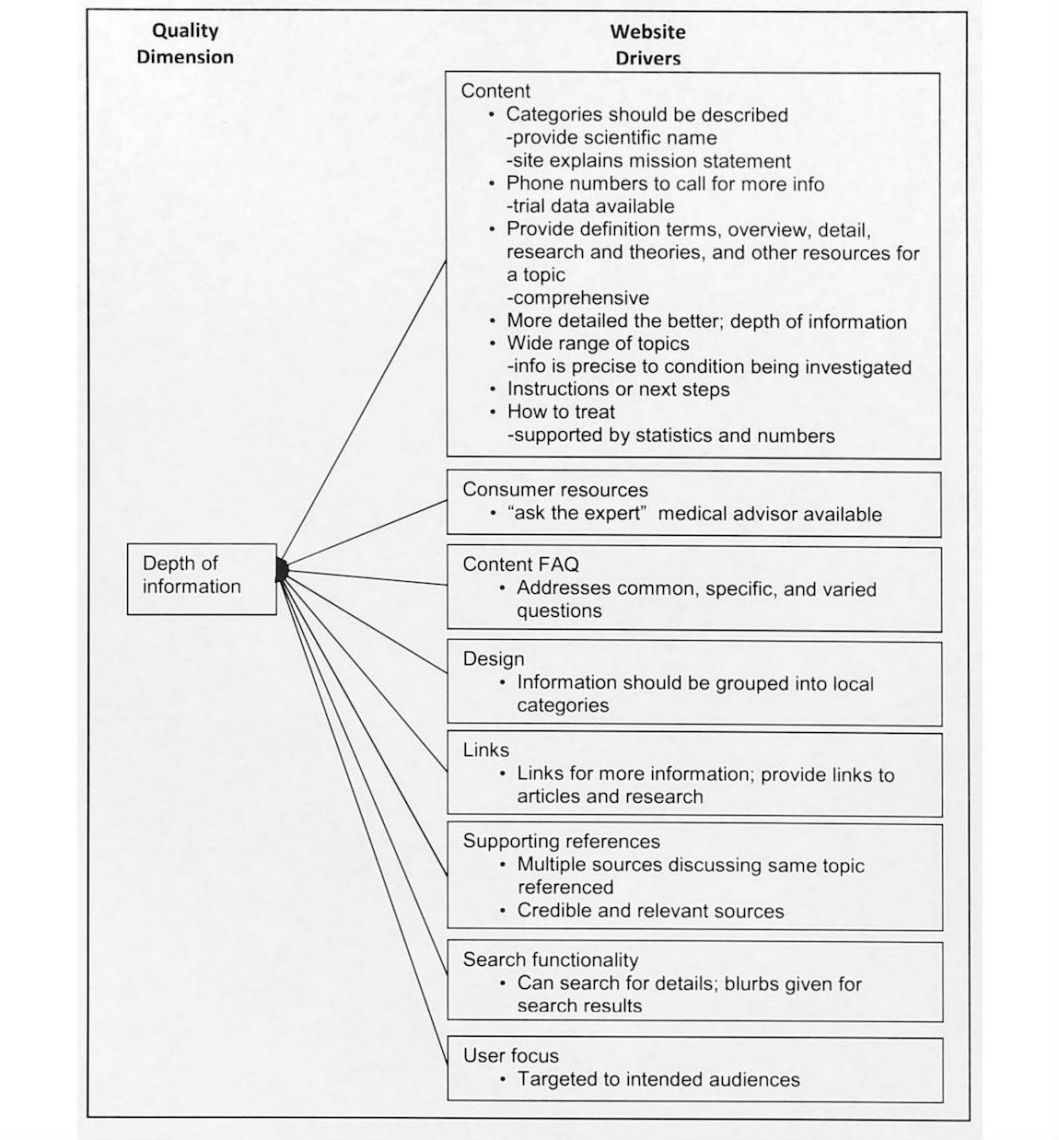
Quality driver themes and detailed drivers for depth of information on health websites.

**Figure 6:**
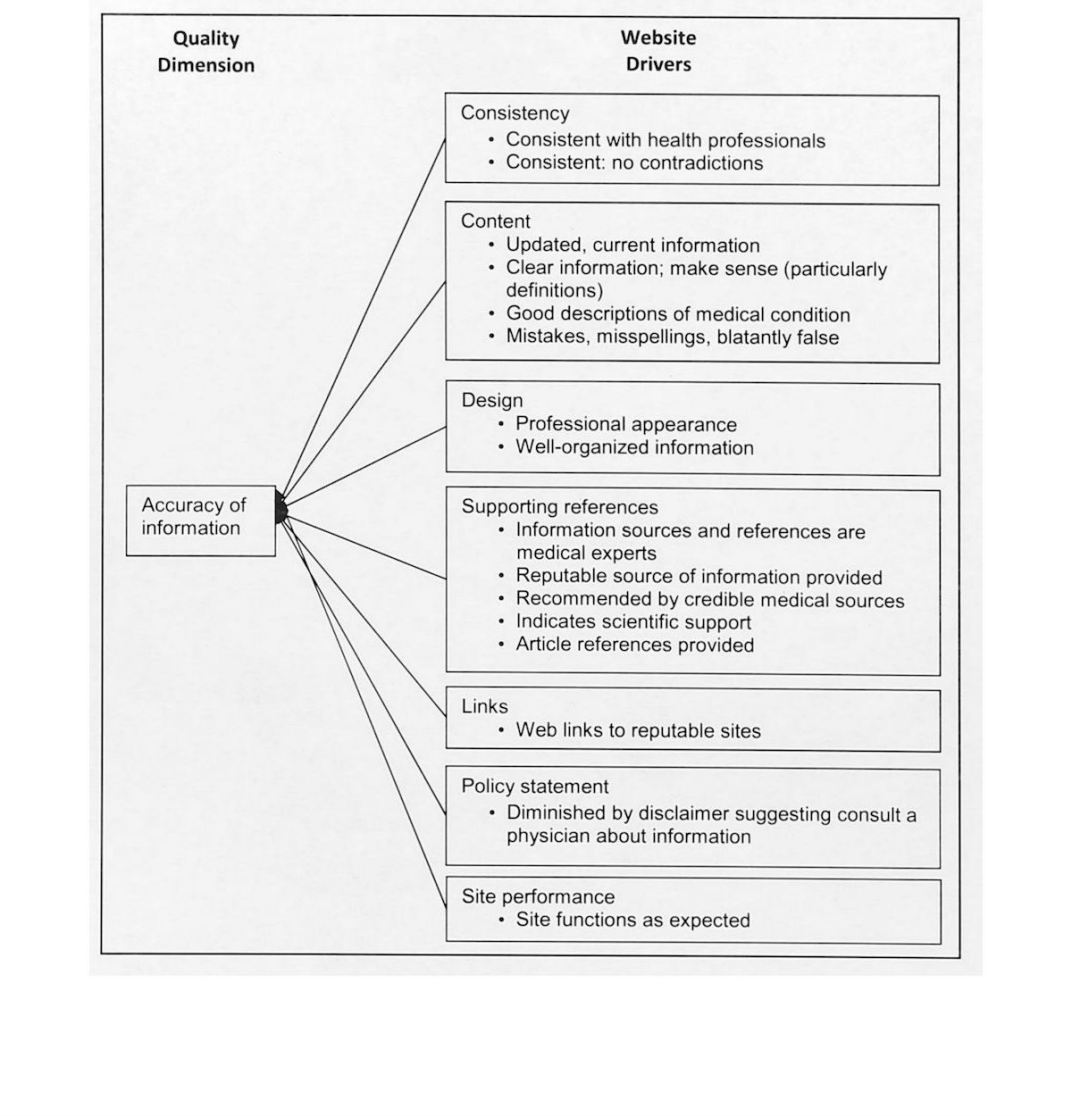
Quality driver themes and detailed drivers for accuracy of information on health websites.

The answer to the qualitative question revealed the meaning of each information quality dimension from the study participant’s perspective. We labeled the meaning of each quality dimension with quality drivers, which indicate the study participants’ perceptions about quality dimensions and tangible website features and functions expected for a health website. Figures 2-6 illustrate the associated supporting drivers (see bullet points in each figure) for each of the five quality dimensions. Collectively, these figures provide a health information quality framework with quality dimensions and drivers targeted at educated young adults.

### Driver Themes Crossing Information Quality Dimensions for Health Websites

We found recurring drivers and grouped them into the driver themes. Figures 2-6 illustrate the associated driver themes with the supporting drivers (see bullet points in each figure) for each of the five quality dimensions. These higher order driver themes (eg *, Content*, *Design*, and *Search functionality*) are supported and grounded with the detailed drivers. These driver themes provide a means to analyze across dimensions. Table 3 details distinct and common themes across dimensions.

**Table 3:** Information quality driver themes across quality dimensions.

Quality driver theme	Completeness of information	Understandability of information	Relevance of information	Depth of information	Accuracy of information
Content	X	X	X	X	X
Consumer resources	X	X		X	
Design	X	X	X	X	X
Links	X	X	X	X	
Open access	X				
Supporting references	X			X	X
Search functionality	X	X	X	X	
User focus	X		X	X	
Advertisements			X		
Content FAQ				X	
Consistency					X
Policy statement					X
Site performance					X

We found that the drivers related to *Content* and *Design* are common drivers across all five dimensions. *Links*, *Search functionality*, *User focused* (targeted toward the health care consumer), *Consumer resources*, and *Supporting references* driver themes appear in three and more dimensions. Some driver themes are unique to a certain dimension, such as *Consistency* and *Policy statement* in Accuracy of information, *Open access* in Completeness of information, *Advertisements* in Relevance of information, and *Content FAQ* in Depth of information.

## Discussion

The study findings lay out a multidimensional and conceptual framework of young educated adults’ perceptions on health website information quality with five quality dimensions and the supporting drivers for each of individual dimensions. In general, few studies of health website information quality make any efforts to compare multiple dimensions of health information quality and *drill down* into the quality driver level [9,18]. We add to the novelty of this study by also identifying general driver themes and discovered recurring themes crossing dimensions.

### Information Quality Dimensions for Health Websites

Although some aspects of the five dimensions of information quality are individually supported in past studies [8-11,20,24,37,38], the collective list has not been represented in any one study or collectively constructed from the ground up with users to our knowledge.

Ideally, all drivers associated with the identified dimensions would be strategically and systematically applied to the health website design to address health information quality dimensions. However, adding features and services to a website are associated with time and cost. Having some indication of priority can help developers make decisions when decisions regarding features, functions, and services must be made to align with time and dollar budgets. On the basis of the study findings, developers may want to pay extra attention to Completeness of information and Understandability of information drivers, given their rated importance by young educated adult consumers. *Consumer resources* drivers such as *provide sources for professional help, provide solution options, and “ask the experts” medical advisor available* may merit special attention, as they are associated with these two quality dimensions.

We acknowledge that Accuracy of information is an important quality dimension in the health care context, as health information could significantly impact consumers’ decisions on treatments [2,6]. Although included in the resulting information quality framework, participants in this study did not rate Accuracy of information as high as the other information quality dimensions (see Table 2). This is an interesting finding, as information accuracy received the highest ratings among competing dimensions in other studies [9,41]. There are a few possible reasons for this finding. First, study participants may possess some underlying assumption that health care experts are the sources of content of health websites who validate the accuracy of information on health websites before being released to general consumers. Second, the study population of young educated adults is Internet savvy and usually has a relatively high eHealth literacy level [69]; therefore, this population may employ their Internet skills to perform cross-validation techniques to assess information accuracy by searching other sites. Third, understandability may at least, in part, serve as a proxy for assessing accuracy; medical terms and descriptions may confuse those not trained in a medical field. The educated young adult consumer group may interpret complete understandable information as possessing greater information quality over information that is complex and challenging to discern, even if accurate. Fourth, as we gave study participants a fairly wide varieties of contexts to answer interview questions and did not ask study participants’ health status during data collection, relevance, depth, and accuracy of information may not be as important as Completeness and Understandability of information depending on the incidents (eg, well or ill) they used at the time of data collection.

This study does not subordinate the need for accurate health websites but does provide indication that health care consumers use comprehension, logic, and easily discerned indicators of mistakes to assess accuracy. The following *Content*-related drivers provide some insight into the attributes that are considered in assessing the accuracy of information: *good description of medical conditions, no misspelling, definitions making sense, and updated or current information,* as well as *consistency, clean design, policy, supporting references* with credible sources *,* and *site performance* to construct perceptions of accuracy. Our findings support that accuracy alone does not result in young educated adults’ perceptions of health information quality; additional quality dimensions are necessary to construct their perceptions of health information quality. The message for website sponsors and designers is two-fold: (1) aim for health care consumers to understand health information with accuracy and (2) engage with the website’s target audience to assess the understandability and perceptions of accuracy.

### Information Quality Drivers for Health Websites

Our study revealed quality drivers associated with the identified dimensions that are specific to the young educated adult consumer group. The drivers associated with Completeness of information and Understandability of information reveal that the young adult population expects that health websites *provide sources for professional help*, provide *solution options*, and make *“ask the experts” medical advisor available.* Our list of drivers also indicate that a certain level of customization is desired as indicated by drivers, such as information *tailored to meet their own need,* and *provide treatment options* and *personalized health tools.*

In contrast to previous studies [10,18,20], our findings did not reveal quality drivers related to the privacy issues. This finding may be attributed to the popularity of social media websites (eg, Facebook, blogs, and Twitter) among young educated adults [33]. Given the openness of social media, young adults may pay more attention to the speedy communication and seek online group support rather than the concerns of protecting their personal health information.

We identified abstract driver themes related to these drivers to provide a means to further analyze the drivers, particularly to determine relationships among dimensions. We found that several driver themes exist in more than one dimension. Different dimensions manifesting the same driver themes indicate some degree of overlap in the conception of dimensions on the websites. The most common driver themes (occurring in three or more of the information quality dimensions) include: *Content, Design, Links, User focus,* and *Search functionality*. We will address the drivers related to these recurring themes for cross-dimensional insights.

Concerning content, previous studies evaluating health information quality found content quality was mostly derived from domain-specific medical guidelines, textbooks, or literature [10,20]. In contrast to the previous studies, our study found that study participants seek more detailed and practical information, such as *descriptions of medical conditions, list of symptoms with an accurate symptom checker, medical stories, terms defined and/or explained, adding pictures, trial data available,* and so on, as the young adults population expect to find actionable advice from the Internet rather than only gaining knowledge about certain health issues. With high education level, young adults have high expectations on the content of health information. For example, young educated adults expect the content to be specific and provide some depth as reflected by the following drivers: *provide research and theories for a topic, instructions or next steps,* and *how to treat supported by statistics and numbers*. Furthermore, young educated adults seem to have an appreciation for varied forms of content, including text, graphics, pictures, audio or videos, animations, and any other form of information presentation [5,18].

It is possible that repeated driver occurrence with the five various dimensions reveals particularly important attributes for designers to consider when prioritizing functionality. The recurring drivers related to the *Design* theme focus on the organization of content, and frequently reference categorization and grouping. The drivers related to *Links* reference connecting to outside websites (perhaps for more information or to validate website information). *User focus*–related drivers reveal the attributes of use a *lay language,* information *tailored to meet their own need, provide treatment options* and *medical advice,* as well as *symptom checker and personalized health tools,* such as providing tailored medical advice based on the information input by health care consumers. A comprehensive search function (across the website) seemed to be a recurring theme with *Search functionality*, which highlights young educated adults’ expectations for the website interactivity features and the efficiency in seeking information.

### Limitations and Future Studies

This study does have limitations in interpretation and generalization, which help to point the direction toward further research. First, although there are comprehensive quality criteria, guidelines, and voluntary codes of trust for both website developers to comply with and for consumers to judge the quality of a website [25,58], no *golden standard* criteria for assessing information quality in health websites from a health care consumer’s perspective have been accepted [11]. The quality dimensions and drivers found in this study set a foundational attempt to provide tangible guidelines of website information quality features for website developers and health care consumers to reference. More studies should be conducted to perfect and validate information quality dimensions and drivers to achieve the model parsimony. A standard instrument that measures consumers’ information quality perceptions needs to be developed with the validated quality drivers explored from this study as items to measure each of the five dimensions, to devise appropriate rating scales, and to test them out and be finally refined. This instrument should be designed to be able to measure perceived information quality in different contexts, such as populations with focused characteristics, purposes, and reasons for seeking health information on the Web, with appropriate changes in wording.

Second, the sample only included individuals with health care backgrounds and business professions and within a certain age span. Although the sample represents two large groups of health care consumers, not all types of potential health care consumers in varying backgrounds are represented. Future work may expand the populations of study and decompose the construct of health care consumers with different characteristics (eg, age, socioeconomic status, education level, and health status and conditions) to investigate the impact of consumers’ perceptions on health website information quality evaluation. An example research question could be “What differences will be in the importance rating of health information quality dimensions by consumer groups with different health literacy levels?”

Third, as the general structure showed in Figure 1, a substantial number of empirical studies have examined the relationship between quality dimensions and higher level evaluations (eg, consumer satisfaction) but not the association of quality drivers (eg, attributes) with quality dimensions as done in this study. Information quality dimensions and drivers identified in this study can be adapted to those causal models to examine how objective quality drivers (ie, attributes) that drive information quality dimensions in health websites impact higher level evaluations. An example research question could be “How do the quality drivers in the *Design* driver theme impact completeness, understandability, relevance, depth, and accuracy of health information, which further impact the use of health websites?”

### Conclusions

This study fills the gap in the consumer health informatics field by defining the quality of health information on health websites through a detailed, multilevel health information quality framework, with dimensions and drivers specified from the perspective of young educated adults. The multidimensional framework of health website information quality presented in this study unifies as well as extends the existing representations of website information quality in the literature. The quality dimensions and drivers found in this study (1) are a first attempt to provide a comprehensive framework specifying underlying meaning of individual quality dimensions, (2) extend existing frameworks by associating these drivers with corresponding quality dimensions, (3) provide a unique view of information quality that has not been specified to such a granular level, and (4) provide a solid foundation for developing an instrument or tool to guide the evaluation of health information from health websites.

Our health website information quality framework has implications for user-centered design and health information system evaluation for the young educated adult audience. It is clear from the findings that accuracy is a foundation, rather than a complete expression of information quality in designing health websites. The identified quality drivers provide indication of what website features young adults consider when they evaluate health website information quality and therefore can be used in research and practice as levers to guide development and assessment of information quality of health websites and to better understand the target group.

We encourage future efforts to validate the proposed framework in additional contexts and with additional user groups. We also encourage using study results as a start toward developing a standard health information quality assessment tool. In addition, we acknowledge that information quality is only one aspect of health website quality. Future research to conceptually decompose other aspects of health website quality, such as design quality, is needed.

## Abbreviations

TAM: Technology Acceptance Model
